# Nursing students’ perception of the clinical learning environment and supervision in relation to two different supervision models – a comparative cross-sectional study

**DOI:** 10.1186/s12912-019-0375-6

**Published:** 2019-10-30

**Authors:** Mirjam Ekstedt, Marléne Lindblad, Anna Löfmark

**Affiliations:** 1 Department of Health and Caring Sciences, Linnaeus University, 392 31 Kalmar, Sweden; 2 Department of Learning, Informatics, Management and Ethics, Karolinska Institutet, Stockholm, Sweden; 3 Department of Health Care Sciences, Ersta Sköndal Bräcke University College, Stockholm, Sweden; 4 Royal Institute of Technology, KTH School of Engineering Sciences in Chemistry, Biotechnology and Health, Stockholm, Sweden; 5 Faculty of Health and Occupational Studies, University of Gävle, Gävle, Sweden

**Keywords:** Clinical learning environment, Supervision, Student-dedicated rooms, Peer-learning

## Abstract

**Background:**

Knowledge concerning nursing students’ experiences of the clinical learning environment and how supervision is carried out is largely lacking. This study compares nursing students’ perceptions of the clinical learning environment and supervision in two different supervision models: *peer learning in student-dedicated units,* with students working together in pairs and supervised by a “preceptor of the day” (model A), and *traditional supervision*, in which each student is assigned to a personal preceptor (model B).

**Methods:**

The study was performed within the nursing programme at a university college in Sweden during students’ clinical placements (semesters 3 and 4) in medical and surgical departments at three different hospitals. Data was collected using the Clinical Learning Environment, Supervision and Nurse Teacher evaluation scale, CLES+T, an instrument tested for reliability and validity, and a second instrument developed for this study to obtain deeper information regarding how students experienced the organisation and content of the supervision. Independent t-tests were used for continuous variables, Mann-Whitney U-tests for ordinal variables, and the chi-square or Fischer’s exact tests for categorical variables.

**Results:**

Overall, the students had positive experiences of the clinical learning environment and supervision in both supervision models. Students supervised in model A had more positive experiences of the cooperation and relationship between student, preceptor, and nurse teacher, and more often than students in model B felt that the ward had an explicit model for supervising students. Students in model A were more positive to having more than one preceptor and felt that this contributed to the assessment of their learning outcomes.

**Conclusions:**

A good learning environment for students in clinical placements is dependent on an explicit structure for receiving students, a pedagogical atmosphere where staff take an interest in supervision of students and are easy to approach, and engagement among and collaboration between preceptors and nurse teachers. This study also indicates that supervision based on peer learning in student-dedicated rooms with many preceptors can be more satisfying for students than a model where each student is assigned to a single preceptor.

## Background

A constructive clinical learning environment, with satisfactory possibilities for student learning, and a focus on student learning needs, is vital to nursing education [1]. Clinical learning is carried out in complex health care settings, and students’ experiences within the clinical context are of great importance to how and what they learn. The environment should motivate students and contribute to their feeling of security, including when asking questions to achieve learning outcomes [2]. The clinical learning environment and supervision of students play a crucial part in supporting student learning and are highly dependent on the relationship between student, preceptor, and nurse teacher [3]. Nurse teachers have a multifaceted and important role in supervision, including supporting, motivating, problem-solving and monitoring [4]. Students have a responsibility to be active in their own learning [5] and preceptors and nurse teachers are both facilitators of and responsible for the students’ learning in clinical settings.

From the students’ perspective, the clinical environment has been described as non-supportive, e.g., because of organisational shortcomings, a lacking relationship between students and preceptors, and negative attitudes and behaviours on the part of preceptors [6], Students have also described inhibitors to learning arising from preceptors’ lack of engagement and feedback [7]. Preceptors were not always engaged personally or easy to reach, and students found that theory and practice were not clearly connected to each other and felt that they lacked opportunities to reflect together with their preceptors [8]. A key challenge reported by preceptors is finding adequate time to supervise students in the clinical setting and this, along with the lack of recognition by both the faculty and health care organisations, seems to undermine the importance of the role [9]. Preceptors have stated that they must strike a balance between taking care of patients and supervising students [10]. There is a risk that the clinical demands may take time from supervision and thus affect student learning [11].

In the search for new innovative pedagogical models in the clinical setting, peer learning is found to be a valuable resource [12]. In a peer learning model described by Pålsson and colleagues [13], students attending a course are divided into pairs, each supervised by one preceptor. The contribution of peers has often been underestimated, but can be a key component of clinical learning that impacts on student experiences [13–16]. When students get the opportunity to assume responsibility for a patient’s care and share their experiences with a peer, learning may increase [14, 16]. Peer learning has also been described to contribute to developing the students’ abilities to communicate and solve problems to a greater extent than a more traditional way of learning, as well as alleviating levels of stress and anxiety [17].

Other innovative ways of developing the learning environment and supervising students include having nurse teachers and preceptors working together in models that enable for clinical wards to effectively supervise large numbers of students. Collaborative models, such as student-dedicated units, have been introduced. The teaching and learning environment at such units is developed through the collaborative efforts of preceptors and nurse teachers. A unit consists of one or two patient rooms, in which peer learning is combined with patient-centred training [18]. Several studies have reported that students have positive experiences of supervision combined with peer learning in student-dedicated units [5, 19, 20]. Students express that they become more confident as they assume increasing responsibility for patients and that they also discover their professional role when they get opportunities to work independently and are given responsibility during their clinical placements [5].

However, comparisons of students’ experiences of their learning and supervision in different models are limited. Today, we are seeing an increasing number of students, and a simultaneous reduction of the number of beds and patients in teaching hospitals, where most clinical placements occur. This calls for the highest possible standard in clinical education, and therefore it is of the utmost importance to investigate and evaluate supervision models that meet these challenges.

Students’ experiences of clinical education are relevant as they impact on the opportunities of linking the theoretical aspect of studies with clinical practice. The nursing student’s satisfaction with the learning environment and the supervisory relationship are suggested as important factors that could be used as a basis for potential development and/or reforms of learning environments in clinical settings [21]. Although theoretical conceptualisation is lacking, there is evidence that nursing students’ satisfaction with the clinical learning environment and supervision could be conceptually described and measured within the context of an internationally validated tool. The Clinical Learning Environment, Supervision and Nurse Teacher (CLES+T) [22] evaluation measure conceptualises the key components in five distinct dimensions: the ward’s pedagogical atmosphere, the leadership style of the ward manager, premises of nursing on the ward, various aspects of the supervisory relationship and the role of the nurse teacher in clinical practice in transforming theoretical knowledge into clinical practice and skills [22, 23]. Therefore, the aim of this study was to compare nursing students’ experiences of the clinical learning environment and the supervisory relationships from two different supervision models used in a Swedish nursing education. The two supervision models are *peer learning in student-dedicated units*, with students working together in pairs and supervised by a preceptor “for the day” (model A), and *traditional supervision*, in which each student shadows a preceptor in her/his work within nursing care (model B).

## Methods

### Design

This cross-sectional study investigates various aspects of two supervision models in a comparative between-subject design, using quantitative data gathered from students through questionnaires. The study adheres to the STROBE methodology for cross-sectional studies.

### Setting

The study took place within the framework of the nursing programme at a university college in Sweden. About 50% of the three-year nursing programme (corresponding to 180 credits in the European Credit Transfer System) consists of clinical education. Students’ clinical placements during the second year (semesters 3 and 4), each a five-week period, were carried out in various medical and surgical departments at three different hospitals (for confidentiality reasons labelled Alpha, Beta, Caesar in Table 1).

**Table 1:** Demographic characteristics and supervision conditions for nursing students

	Model A (*N* = 170)	Model B (*N* = 74)	*p* value
*Gender, N (%)*			0.651
Female	152 (90)	63 (88)	
Male	17 (10)	9 (12)	
Age, median (range)	26 (21–50)	25 (21–51)	0.262
Age, mean (SD)	28 (6)	28 (7)	
*Study semester, N (%)*			0.577
Semester 3	77 (45)	37 (50)	
Semester 4	93 (55)	37 (50)	
*Hospital setting, N (%)*			< 0.001
Alpha	13 (8)	1 (1)	
Beta	32 (19)	62 (84)	
Caesar	125 (73)	11 (15)	
More than one preceptor, N (%)	153 (90)	50 (68)	< 0.001
No of preceptors, median (range)	6 (1–13)	2 (1–8)	
No of preceptors, mean (SD)	6 (2)	3 (2)	

Supervision during the clinical placements was provided by preceptors. Nurse teachers employed by the university college had an overall responsibility for the clinical education in both supervision models. A nurse teacher met each student and his/her preceptor twice during the five-week period of clinical education in order to clarify objectives and learning outcomes and to discuss the student’s progress. The nurse teachers’ responsibilities, which include assessment and grading of the students’ achievements, are explicitly described by Kristofferzon and colleagues [24].

### Supervision models

Supervision was carried out in two different supervision models. Both models were used in parallel, but on different wards, at the three hospitals. Students in model A worked in pairs in student-dedicated units and were supervised by “the preceptor of the day,” while those in model B had traditional supervision, where each student was assigned to a personal preceptor and followed mainly this person in daily patient care. Model A, which has been introduced at a number of wards at the three hospitals (Table 1), was guided by peer-learning strategies [12, 18, 25] and patient-centred care, which in this study meant that the students followed the patients, not the preceptor. A student pair had continuous responsibility for all the nursing care given to “their” patients in the student-dedicated unit, throughout the clinical placement. The preceptor was responsible for supervision of one pair of students at a time, offering them supervision and exchanging thoughts with them. Supervision model B, the traditional model, where one student is assigned to a personal preceptor and his/her patient care [26], was still used on a number of wards at the three hospitals. The preceptor could change from 1 day to the next in both models, depending on work organisation at the ward, sick leave or other unexpected occurrences.

### Measures

The Swedish version of the Clinical Learning Environment, Supervision and Nurse Teacher (CLES+T) evaluation scale [22] was used to measure the students’ perceptions of the clinical learning environment and the supervisory relationship during clinical education. A questionnaire was used to gather demographic data on student age, which semester and clinical placement they were in, and the number of preceptors during their clinical placement. A supplementary questionnaire was used to obtain additional information about each student’s satisfaction with the preceptor’s role and their own preparedness for clinical supervision and professional progress. CLES+T has been tested for reliability and validity among Swedish nursing students during clinical placement at hospitals [23, 27], with Cronbach’s alpha values ranging from 0.75 to 0.96 [23]. The 34 statements in CLES+T are divided into five sub-dimensions: “Pedagogical atmosphere on the ward” (nine items), “Leadership style of the ward manager” (four items), “Premises of nursing on the ward” (four items), “Supervisory relationship” (eight items) and “Role of the nurse teacher during the clinical placement” (nine items). Responses to CLES+T are given on a five-point Likert scale with the following alternatives: (1) fully disagree; (2) disagree to a certain extent; (3) neither agree nor disagree; (4) agree to a certain extent; (5) fully agree. In the current study, the Cronbach’s coefficient alpha was calculated for internal consistency of the total scales, as well as for the sub-dimensions used in the analysis. The alpha value for CLES+T was 0.95 for the total scale, and ranged from 0.75 (premises of nursing on the ward) to 0.96 (supervisory relationship) for the five sub-dimensions.

The supplementary questionnaire was originally developed by the last author (AL), based on her experiences as a faculty teacher, and used for evaluation of the clinical nursing education. The content validity is based on 21 interviews with a group of colleagues, who were asked to consider aspects of the contents, such as clarity, coverage, relevance, and wording of each question. To examine the content validity, experts familiar with the construct of interest and/or experts on the research subject reviewed all of the questionnaire items for readability, clarity, relevance and comprehensiveness. Then, appropriate modifications were made and the final questionnaire was developed. A factor analysis demonstrated that the items loaded on four factors. Based on theoretical reasoning the final questionnaire was modified to three subscales, which consisted of 20 statements regarding “preparedness of student and ward for supervision” (six statements), “the preceptor’s role” (seven statements) and “the student’s professional progress” (seven statements). The respondents were asked to score how well each statement matched their perceptions, using a four-point Likert scale: (1) not at all, (2) to a fairly small degree, (3) to a fairly high degree, and (4) to a very high degree. The Cronbach’s alpha for the supplementary questionnaire was 0.86 for the total, and ranged from 0.71 to 0.76 for the subscales, showing moderate internal consistency. The items and subscales are shown in Table 3.

### Procedures and sample characteristics

This study was conducted during three semesters: spring and fall 2011 and spring 2012. All students in the second year (semesters 3 and 4) of the nursing programme got verbal and written information about the study aims and procedures. The students were offered clinical placements by a university coordinator, who was not involved in teaching, on a ward with either supervision model A or supervision model B. This was based on the regular procedure for coordinating clinical placements. The preceptor introduced the students to the routines on the ward and the supervision model for their clinical practice, regardless of which model was used. Nurse teachers handed out questionnaires during a lesson following completion of the clinical placement. The students were explicitly informed that participation was voluntary and that it would not affect their education if they declined to participate. They were assured confidentiality and informed that the results would be presented at the group level. The students who decided to participate returned the questionnaire together with informed consent to the nurse teachers in a closed envelope addressed to the first author, or put it in the mailbox of one of the teachers.

### Data analysis

The statistical analyses were carried out using the Statistical Program for the Social Sciences (SPSS ver. 25). Descriptive statistics were presented as mean and standard deviations or median (range) for numerical variables, or as frequency or percentages for categorical variables. Independent t-tests were used for continuous variables, Mann-Whitney U-tests were used for ordinal variables or when there was violation of assumption for continuous variables and chi-square or Fischer’s exact tests were used for categorical variables. The statistical significance level was set to 5% (alpha = 0.05). The effect size calculation for group mean differences was based on Hedges’ g, as the sample size differed between the two groups. Hedges’ g [28] provides a measure of effect size weighted according to the relative size of each sample. The effect size for ordinal variables where we employed Mann-Whitney’s test was calculated using the equation from Rosenthal [29], and interpreted using Cohen’s recommendation: 0.2 = small; 0.5 = medium; 0.8 = large. Cronbach’s coefficient alpha was calculated for internal consistency of the total scales as well as for the sub-dimensions used in the analysis. The estimate of reliability, Cronbach’s alpha, should exceed 0.7.

### Ethical considerations

The head of the department at the university granted permission to perform the study. The researchers were not involved in teaching or grading of the students participating in this study. The questionnaires were coded and only the researchers had access to the coding list. This study followed the ethical requirements stated in the Declaration of Helsinki. As this study does not involve patients or relatives or sensitive personal information, no ethical approval was required under the Swedish Act concerning the Ethical Review of Research Involving Humans, from the Ministry of Education and Research [30].

## Results

Out of 381 eligible students, 244 filled out questionnaires, yielding a response rate of 64%. Of these, 170 students (90% women) got supervision in model A and 74 students (88% women) in model B. The students’ mean age was 28 years and the range was between 21 and 50/51 years in both groups. There was no difference between the groups in the proportion of students who were in semesters 3 and 4, respectively (Table 1). However, there was a significant difference between the three hospital settings as regards the organisation of supervision. In wards using model A, 90% of students received supervision from more than one preceptor, while in those using model B, the proportion of students supervised by more than one preceptor was 68%.

### The clinical learning environment

Overall, the student nurses had positive experiences of the clinical learning environment in both supervision models. The ratings of the three sub-dimensions “pedagogical atmosphere,” “leadership style of the ward manager” and “premises of nursing on the ward” had mean values between 3.1 and 4.5 (Table 2). While there were no substantial differences between groups for any of the three sub-dimensions, there were differences regarding their ratings on single items in favour of model A. These items concerned if the ward was regarded as a good learning environment, and if staff were easy to approach and generally interested in supervising students.

**Table 2:** Students’ experiences of the clinical learning environment (CLES+T) in the two supervision models

	Model A (*n* = 170) Mean (SD)	Model B (*n* = 74) Mean (SD)	*p* value	Effect size^a^
Pedagogical atmosphere (alpha = 0.89)	**4.2 (0.6)**	**4.0 (0.7)**	**0.107**	**0.32**
1.Staff were easy to approach	4.3 (0.8)	4.0 (1.0)	0.027	
2. I felt comfortable going to the ward at the start of my shift	4.3 (0.9)	4.1 (1.0)	0.067	
3. During staff meetings (e.g., before shifts), I felt comfortable taking part in the discussion	3.6 (1.0)	3.6 (1.0)	0.791	
4. There was a positive atmosphere on the ward	4.2 (0.9)	4.2 (0.8)	0.485	
5. Staff were generally interested in supervising students	4.0 (0.9)	3.7 (1.0)	0.020	
6. Staff knew each student by first name	4.0 (1.0)	4.0 (1.1)	0.607	
7. There were sufficient meaningful learning situations on the ward	4.3 (0.8)	4.3 (0.7)	0.610	
8. The learning situations were multi-dimensional in terms of content	4.2 (0.8)	4.1 (0.9)	0.725	
9. The ward could be regarded as a good learning environment	4.5 (0.9)	4.2 (0.9)	0.003	
Leadership style of the ward manager (WM) (alpha = 0.85)	**3.6 (0.9)**	**3.8 (0.9)**	**0.418**	**0.22**
10. The WM regarded staff on her/his ward as key resources	4.2 (0.8)	4.1 (0.9)	0.924	
11. The WM was a team member	3.7 (1.1)	3.8 (1.2)	0.216	
12. Getting feedback from the WM could easily be regarded as a learning situation	3.1 (1.2)	3.4 (1.2)	0.126	
13. The efforts of individual employees were appreciated	3.6 (1.0)	3.7 (1.1)	0.624	
Premises of nursing on the ward (alpha = 0.75)	**3.8 (0.7)**	**3.9 (0.7)**	**0.218**	**0.15**
14. The ward’s nursing philosophy was clearly defined	3.3 (1.0)	3.5 (1.0)	0.337	
15. Patients received individual nursing care	4.2 (0.8)	4.1 (0.9)	0.510	
16. There were no problems in the information flow related to patient care	3.9 (1.0)	4.0 (0.9)	0.419	
17. Documentation of nursing (e.g., nursing plans, daily recording of procedures) was clear	3.9 (1.0)	4.1 (1.0)	0.093	
Supervisory relationship (alpha = 0.96)	**4.3 (0.8)**	**4.3 (0.9)**	**0.360**	**0.0**
18. My preceptor showed a positive attitude towards supervision	4.5 (0.7)	4.3 (1.0)	0.304	
19. I felt that I received individual supervision	4.2 (0.9)	4.4 (1.0)	0.066	
20. I continuously received feedback from my preceptor	3.9 (1.0)	4.0 (1.2)	0.424	
21. Overall, I am satisfied with the supervision I received	4.4 (0.9)	4.3 (1.1)	0.735	
22. The supervision was based on a relationship of equality and promoted my learning	4.2 (0.9)	4.3 (1.1)	0.327	
23. There was mutual interaction in the supervisory relationship	4.3 (0.8)	4.3 (1.0)	0.384	
24. Mutual respect and approval prevailed in the supervisory relationship	4.4 (0.9)	4.4 (1.0)	0.210	
25, The supervisory relationship was characterised by a sense of trust	4.4 (0.9)	4.4 (1.0)	0.383	
The role of the nurse teacher (NT) in clinical practice (alpha = 0.87)	**3.9 (0.7)**	**3.6 (0.8)**	**0.003**	**0.41**
26. In my opinion, the NT was capable of integrating theoretical knowledge with the everyday practice of nursing	4.2 (0.7)	4.1 (0.7)	0.333	
27. The NT was capable of operationalising the learning goals of this clinical placement	4.1 (0.8)	4.0 (0.8)	0.518	
28. The NT helped me reduce the theory-practice gap	4.0 (1.0)	4.0 (0.9)	0.848	
29. The NT was like a member of the nursing team	3.2 (1.4)	2.6 (1.4)	0.006	
30. The NT was able to impart his or her pedagogical expertise to the clinical team	3.4 (1.2)	2.9 (1.3)	0.009	
31. The NT and the clinical team worked together supporting my learning	3.9 (1.1)	3.2 (1.4)	< 0.001	
32. The meetings between myself, the preceptor and the NT were a pleasant experience	4.4 (0.8)	4.0 (1.0)	0.004	
33. The atmosphere at the meetings was congenial	3.4 (1.1)	2.9 (1.4)	0.007	
34. The focus of the meetings was on my learning needs	4.2 (0.9)	4.2 (0.9)	0.345	

*Items rated on a five-point Likert scale ranging from: 1 (not at all/disagree entirely) to 5 (agree entirely), tested with Mann-Whitney U test.* ^*a*^ *Effect size calculated with Hedges’ g. Missing data in Model B ranged from 0 to 10; missing data in Model A ranged from 10 to 21*

### The supervisory relationship

There were no differences between the two supervision models within the subject area “supervisory relationship” and the ratings were high overall in both models (range 3.9 to 4.5). However, students supervised in model A gave higher ratings for the single item “I felt that I received individual supervision” than students supervised in model B (Table 2).

### The role of the nurse teacher during the clinical placement

Ratings showed a substantial difference between the two supervision models in the subject area “the role of the nurse teacher” (*p* = 0.003). The student ratings in this area were significantly more in favour of supervision with model A for five out of nine items (Table 2). Student ratings within the sub-areas “cooperation between placement staff and nurse teacher” and “relationship between the student, preceptor and nurse teacher” resulted in significant differences, where students supervised in model A had more positive experiences. Students supervised in model A to a greater extent than students in model B experienced that the nurse teacher was an integrated part of the nursing team, was able to impart expertise to the team and that the team worked together supporting student learning. Students in model A also had more positive experiences of the relationship between the student, preceptor and nurse teacher than students in model B, stating that meetings were generally a pleasant experience in a congenial atmosphere. The three items concerning the nurse teacher “enabling integration of theory and clinical training” did not reveal any differences between the groups.

### Preparedness for supervision

Nursing student ratings on statements in the second questionnaire showed that they were, in general, satisfied with their own preparedness and the ward’s preparedness for supervision. The most significant differences with respect to how supervision was organised concerned the clarity and structure of the supervision; students in model A to a greater extent than students in model B experienced that there was an explicit structure for receiving students, that the ward had an explicit model for supervising students, and that the ward had resources (i.e., personnel) dedicated to supervision of nursing students (Table 3).

**Table 3:** Students’ satisfaction with supervision, preceptor’s role and professional progress in the two supervision models

		Model A N (%)	Model B N (%)	*p* value	Effect size^a^
Preparedness for supervision (alpha = 0.71)				**0.000**	**0.29**
I was adequately prepared for the clinical placement	not at all fairly small degree fairly high degree very high degree	0 (0.0%) 12 (7.1%) 127 (75.1%) 30 (17.8%)	1 (1.4%) 9 (12.3%) 43 (58.9%) 20 (27.4%)	0.043	
My knowledge about the expected learning outcomes was adequate	not at all fairly small degree fairly high degree very high degree	0 (0.0%) 15 (8.8%) 114 (67.1%) 41 (24.1%)	0 (0.0%) 7 (9.5%) 45 (60.8%) 22 (29.7%)	0.619	
The ward had dedicated resources for supervision	not at all fairly small degree fairly high degree very high degree	1 (0.6%) 12 (7.1%) 41 (24.4%) 114 (67.9%)	2 (2.7%) 12 (16.2%) 29 (39.2%) 31 (41,9%)	0.001	
There was an explicit structure for receiving students	not at all fairly small degree fairly high degree very high degree	5 (3.0%) 9 (5.3%) 42 (24.9%) 113 (66.9%)	3 (4.1%) 18 (24.3%) 30 (40.5%) 23 (31.1%)	0.000	
The ward could be regarded as a good learning environment	not at all fairly small degree fairly high degree very high degree	1 (0.6%) 8 (4.7%) 36 (21.2%) 125 (73.5%)	1 (1.4%) 7 (9.5%) 28 (37.8%) 38 (51.4%)	0.009	
The ward had an explicit model for supervising students	not at all fairly small degree fairly high degree very high degree	3 (1.8%) 26 (15.5%) 73 (43.5%) 66 (39.3%)	4 (5.5%) 29 (39.7%) 29 (39.7%) 11 (15.1%)	0.000	
The preceptor’s role (alpha = 0.76)				**0.161**	**0.10**
My identity as a nurse has been reinforced	not at all fairly small degree fairly high degree very high degree	1 (0,6%) 10 (5,9%) 64 (37,6%) 95 (55,9%)	1 (1,4%) 6 (8,1%) 28 (37,8%) 39 (52,7%)	0.837	
The preceptor made room for reflection	not at all fairly small degree fairly high degree very high degree	5 (3.0%) 38 (22.8%) 63 (37.7%) 61 (36.5%)	6 (8.1%) 19 (25.7%) 32 (43.2%) 17 (23.0%)	0.092	
The preceptor gave feedback when tasks were completed	not at all fairly small degree fairly high degree very high degree	5 (3.0%) 38 (22.5%) 72 (42.6%) 54 (32.0%)	4 (5.5%) 14 (19.2%) 30 (41.1%) 25 (34.2%)	0.743	
The preceptor encouraged my asking questions	not at all fairly small degree fairly high degree very high degree	3 (1.8%) 18 (10.6%) 66 (38.8%) 83 (48.8%)	2 (2.7%) 8 (10.8%) 22 (29.7%) 42 (56.8%)	0.564	
The preceptor showed an interest in my studies and exam tasks	not at all fairly small degree fairly high degree very high degree	9 (5.4%) 59 (35.1%) 75 (44.6%) 25 (14.9%)	4 (5.5%) 25 (34.2%) 24 (32.9%) 20 (27.4%)	0.112	
It is beneficial to have several preceptors during a teaching period	not at all fairly small degree fairly high degree very high degree	13 (7.7%) 38 (22.5%) 74 (43.8%) 44 (26.0%)	8 (11.9%) 22 (32.8%) 20 (29.9%) 17 (25.4%)	0.148	
More than one preceptor contributed to the assessment of my learning outcomes	not at all fairly small degree fairly high degree very high degree	14 (8.9%) 23 (14.6%) 67 (42.7%) 53 (33.8%)	18 (27.7%) 13 (20.0%) 19 (29.2%) 15 (23.1%)	0.001	
Professional progress (alpha = 0.76)				**0.000**	**0.30**
My independence has increased	not at all fairly small degree fairly high degree very high degree	0 (0.0%) 1 (0.6%) 62 (36.5%) 107 (62.9%)	0 (0.0%) 4 (5.5%) 29 (39.7%) 40 (54.8%)	0.036	
My capacity for critical thinking has increased	not at all fairly small degree fairly high degree very high degree	0 (0.0%) 6 (3.5%) 84 (49.4%) 80 (47.1%)	0 (0.0%) 1 (1.4%) 40 (54.8%) 32 (43.8%)	0.540	
My problem-solving ability has improved	not at all fairly small degree fairly high degree very high degree	0 (0.0%) 10 (5.9%) 102 (60.0%) 58 (34.1%)	0 (0.0%) 9 (12.5%) 38 (52.8%) 25 (34.7%)	0.194	
I have attained the learning outcomes of the course	not at all fairly small degree fairly high degree very high degree	0 (0.0%) 2 (1.2%) 89 (53.3%) 76 (45.5%)	0 (0.0%) 2 (2.7%) 46 (63.0%) 25 (34.2%)	0.211	
I got a comprehensive picture of the patients during my clinical placement	not at all fairly small degree fairly high degree very high degree	0 (0.0%) 3 (1.8%) 45 (26.5%) 122 (71.8%)	0 (0.0%) 5 (7.0%) 25 (35.2%) 41 (57.7%)	0.031	
Collaboration with peers developed my ability of constructive problem-solving	not at all fairly small degree fairly high degree very high degree	5 (2.9%) 12 (7.1%) 68 (40.0%) 85 (50.0%)	7 (11.1%) 20 (31.7%) 29 (46.0%) 7 (11.1%)	0.000	
Collaboration with peers developed my ability to reflect on different care situations	not at all fairly small degree fairly high degree very high degree	4 (2.4%) 7 (4.1%) 58 (34.1%) 101 (59.4%)	7 (11.1%) 13 (20.6%) 23 (36.5%) 20 (31.7%)	0.000	

^a^Effect size based on Mann-Whitney test results

### The preceptor’s role

Students supervised in model A had more positive experiences of the preceptor’s role and gave higher ratings for the items “it is beneficial to have several preceptors during a teaching period” and “having more than one preceptor contributed to the assessment of my learning outcomes,” as compared with students in model B (Table 3). There were no differences between the two supervision models in regards to preceptors’ encouragement of questions or making room for reflections (Table 3).

### Students’ professional progress

As regards their own professional progress during clinical placement, students supervised in model A perceived that they had developed independence and that they got a “comprehensive picture of the patients during the clinical placement” to a greater extent than students assigned to model B (Table 3). There were no differences in the students’ experiences of developing problem-solving skills or achieving the learning outcomes of the course. For the two items concerning experiences of learning from peers, there were significant differences between students in the different models. Students in model A indicated, to a greater extent than students in model B, that they had increased their ability to solve problems and reflect on various patient care situations in collaboration with peers (Table 3).

## Discussion

This study is one of the few that has compared nursing students’ experiences of different supervision models. The main findings were that both models yielded overall satisfaction among students concerning the learning environment (pedagogical atmosphere, leadership style of the ward manager, premises of nursing on the ward) and the supervisory relationship, as measured with CLES+T. However, model A was rated significantly higher by students than traditional supervision, model B, on items indicating that the ward was a good learning environment, and that staff were generally easy to approach and interested in supervising students.

The number of preceptors per student varied largely in both model A, where students had a designated “preceptor of the day,” and model B, where the preceptor could change from 1 day to another. Students supervised in model A, to a greater extent than those supervised in model B, perceived it was beneficial to have more than one preceptor. This is in contrast with a study by Sundlers et al. [31], also using the CLES+T scale, where the clinical learning environment was studied in relation to three models of supervision. They found that students who had the same preceptor all the time were more satisfied with their relationship with the preceptor than those who had different preceptors each day, whether they followed patients in rooms specifically designated for student training and had a number of preceptors or followed a designated personal preceptor, but also received supervision from others [31]. It was not stated if the students worked in pairs in the student-dedicated rooms. Omer et al. [32] explored and compared students’ perceptions of two supervision models. One model was characterised by intensive preceptorship and the other by increasing independence and self-directed learning. The model with intensive preceptorship was perceived as more satisfactory, which according to the authors could be due to the students not being mature enough to act autonomously for their patients’ interests. However, comparisons of studies in an international perspective are precarious, because of large variations between countries in the organisation of supervision and in preceptors’ roles and responsibilities.

Most of the items concerning the wards’ preparedness and organisation of supervision, the preceptor’s role, and students’ professional progress were rated higher by students who had been supervised in model A. The area “professional progress” included some questions about collaboration with peers. Students who had experiences of working continuously with a fellow student rated items on collaboration and impact on problem-solving ability and reflection higher than students without such experiences. The literature reviews by Secomb [12] and by Stone et al. [17] underline that peer learning has many benefits, e.g., students have described how they discuss and reflect as well as identify and solve problems together. Students in model B were fairly positive to collaborating with peers, although this model does not presuppose a structure with students in pairs all the time.

Students in model A found that they were given the opportunity to work autonomously and to discuss and reflect on care situations with peers, and they experienced, to a greater extent than those in model B, that they developed independence and got a comprehensive picture of patients during their clinical placement. This is in line with other studies where similar supervision models are evaluated [5, 16, 18]. In the study by Hellström-Hyson et al. [5], students appreciated the opportunity to identify and solve problems on their own, with an experienced preceptor within reach. Loke and Chow [25] found that students’ personal development and learning skills grew through peer learning. Thus, being able to cooperate with peers during clinical placement seems to encourage critical reflection and independent thinking, which is important for developing deeper knowledge.

Negative aspects of peer learning and the number of preceptors in the patient-centred supervision models have also been discussed in earlier studies. Loke and Chow [25] described that negative experiences of peer learning were related to having many different preceptors involved in supervision. Sundler et al. [31] concluded in their study that student dissatisfaction with the learning environment was not only associated with having many preceptors, but also with the preceptors’ attitudes and approaches, as well as with having a newly graduated nurse as preceptor. We found, in contrast, that students in model A experienced a greater advantage from having more than one preceptor than students in model B, and also that they felt that having several preceptors contributed to the assessment of their learning outcomes. Much like Trede et al. [33], we argue for shifting from a focus on individual supervision to a focus on collective responsibility for creative workplace environments to promote student learning.

Generally speaking, all students in the current study were satisfied with the relationships with their preceptors. The relationships were equal and based on mutual understanding. Whether students were involved in model A or B, they were encouraged to use reflection as a tool for learning. No difference was found between the groups in their perception of the preceptor’s role in making room for reflection and questions, which may indicate that reflection is used as a valuable tool for feedback and for translating theory into practice [34, 35].

The important role of the nurse teacher, e.g., as a pedagogical expert, was also underlined in this study. This is supported by a number of studies, which have highlighted that the student-teacher relationship is crucial to learning [32, 36]. Studies based on the students’ perspective have also emphasised the important role that visits from the academic nurse teacher play in bridging the theory-practice gap during student placements and helping students reflect on clinical practices [24]. The role also includes supporting fulfilment of theoretical assignments and clarifying the learning outcomes of the course to both the preceptor and the student. Papastavrou et al. [21] have emphasised the role of the nurse teacher in influencing the entire nursing staff to be involved in the students’ learning process.

In this study, the role of the preceptor was described as more prominent on wards where students were supervised with peer learning compared with wards using traditional supervision. The reason for this is unclear, but the nurse teacher was experienced more as a member of the nursing team (preceptor and nurse teacher), able to impart his or her pedagogical expertise, on wards where students were supervised with the patient-centred, peer-learning model. The preceptor and nurse teacher also collaborated in their support of the students’ learning in such a way that the encounters between the student, the preceptor and the nurse teacher were experienced as relaxed and congenial by the student (Table 2). Our study also shows that the wards, in model A, to a greater extent dedicated resources to supervision, and that students experienced that the wards had a clear structure for receiving students and an explicit model facilitating supervision. Altogether, this might have contributed to a closer cooperation between the academic nurse teacher and the preceptors, and contributed to the students’ positive experience of the learning environment. The literature indicates that there is an increased need for university teachers to be available regularly, to both students and staff [37], irrespective of supervision model. Calpin-Davies [38] emphasised that teachers have a distinct advantage over preceptors in helping students, as they are highly aware of the students’ stage of learning, and can adjust teaching and explanations accordingly. Students in the study by Löfmark et al. [39] gave higher ratings for university teacher supervision than preceptor supervision, which might be explained by the teachers’ familiarity with learning outcomes for clinical placements and how they can be achieved and assessed.

### Methodological considerations

This study was methodologically innovative and one strength was that the students’ experiences of two supervision models were possible to gather and compare. Furthermore, using two questionnaires, where one of them is a commonly used, validated and reliability-tested instrument for capturing students’ experiences of their learning environment and supervision, can be seen as a strength. However, the psychometrics of the second questionnaire have not been tested, which limits the value of the additional knowledge of supervision and peer learning it provided. A limitation of this study is the sample size, which makes generalisation problematic. Quantitative methods limit the possibility to extract more detailed information about student experiences regarding the differences between the models of supervision.

## Conclusions

A good learning environment for students in clinical placements is dependent on engagement and collaboration between preceptors and academic nurse teachers. This study also indicates that a supervision model for students based on peer learning in student-dedicated rooms with many preceptors can be more satisfying than a model with traditional supervision, where each student is dependent on a single preceptor. A particularly good learning environment is found on wards with an explicit structure for receiving students, offering supervision from more than one preceptor, and a pedagogical atmosphere where staff are generally interested in supervising students and easy to approach. In the search for the best quality in clinical education, supervision models must be systematically evaluated and compared in order to meet future needs and challenges. Longitudinal evaluation of the effect on students’ learning outcomes is needed.

## Data Availability

The datasets used and/or analysed during the current study are available from the corresponding author on reasonable request.

## Abbreviations

CLES+T: Clinical Learning Environment, Supervision and Nurse Teacher
model A: peer learning in student-dedicated units, with students working together in pairs and supervised by a preceptor “for the day”
model B: traditional supervision, in which each student shadows a preceptor in her/his work within nursing care
